# Major unsolved points in adult neurogenesis: doors open on a translational future?

**DOI:** 10.3389/fnins.2014.00154

**Published:** 2014-06-11

**Authors:** Paolo Peretto, Luca Bonfanti

**Affiliations:** ^1^Neuroscience Institute Cavalieri OttolenghiOrbassano, Italy; ^2^Department of Life Sciences and System Biology, University of TurinTorino, Italy; ^3^Department of Veterinary Sciences, University of TurinTorino, Italy

**Keywords:** brain repair, neurodegenerative diseases, regenerative medicine, therapeutic approaches, neural stem cells, parenchymal progenitors, cell therapy, brain evolution

## Abstract

In spite of many data gathered during the last two decades on adult neurogenesis (AN) it is evident that such knowledge is not sufficient for granting translational outcomes in brain repair, especially if the ultimate goal is to promote cell replacement. Alternative strategies aimed at fostering AN physiological functions (restorative approaches) are still undefined. By asking the question whether AN research field has to be considered as a dead end in the context of brain repair, here we review some unresolved issues: multifaceted evolutionary constraints in mammals, stem/progenitor cell type/availability and tissue permissivity, impact on other brain functions, interplay with other forms of plasticity, and relevance in humans. We suggest that full understanding of AN biology is an essential step for its possible exploitation in brain repair, and that further fundamental, multidisciplinary research is required to reach translational outcomes. Scientist's attitude and their communication skills are also important. To avoid overestimation of AN reparative potential in a translational perspective, more distant goals of cell replacement should be kept clearly distinct from restorative approaches involving AN functional plasticity.

Two decades of investigation on adult neurogenesis (AN) yielded an utterly new vision of brain plasticity and opened new perspectives for brain repair/regeneration strategies. Nevertheless, the ultimate goal of exploiting neurogenic processes for obtaining cell replacement is still far from being achieved. Starting from this antinomy, the big question is: should be the AN research field considered as a dead end in the perspective of brain repair, or, alternatively, is it worthwhile to put in place further efforts in order to solve the problem? By reading the scientific literature, it is clear that all neurobiologists, even believing in an AN translational future, do not share the same answer. Non-univocal visions are normal in a field that has developed by progressively ramifying in many directions accordingly to the different goals pursued by each research group. Some scientists primarily deal with AN physiological roles/mechanisms, apparently being less interested in direct translation of results. Others are mainly focused on aspects that implement AN, or directly address the issue of injury-induced, reactive neurogenesis, paying less attention to the peculiar limits of the mammalian CNS in repairing damage. New translational perspectives in “restorative” rather than “structural reparative” neurology have been recently raised, what could be useful in slowing down the impact of various neurologic impairments (e.g., those occurring in neurodegenerative, vascular, traumatic diseases, age-related cognitive decline), even in the absence of cell replacement. Nevertheless, it is evident that knowledge gathered during the last two decades is not yet sufficient for granting translation of basic neurobiological research. Such inability is linked to several unresolved issues in both physiological and lesion-induced neurogenesis, and to scarcely integrated views between different approaches used to address AN studies. In other words, even in the absence of current, effective therapeutic outcomes, we may not be at a dead end, rather we are in the middle of a route with many new “perfectly reasonable deviations from the beaten track” (Feynman, 2005).

## The present knowledge in mammals: some lights in the dark

Our knowledge of AN in mammals might be grouped in two domains: first, some facts and concepts which are definitively accepted and substantially understood by the scientific community (“acquired knowledge”), and second, a number of issues which remain largely obscure and/or underestimated (“gaps of knowledge”). The main blocks of acquired knowledge can be summarized as follows: (i) two canonical neurogenic zones (subventricular zone, SVZ, and subgranular zone, SGZ) harboring stem cell niches provide neural cell addition into the olfactory bulb and hippocampus (Ming and Song, 2011); we know a lot about their anatomical organization and functional regulation as well as the integration of the newly born neurons (Fuentealba et al., 2012; Tong and Alvarez-Buylla, 2014; Vadodaria and Gage, 2014); (ii) wide areas of the central nervous system (CNS) out of the canonical neurogenic sites host cycling and/or quiescent progenitors which give rise to different processes of non-canonical cell genesis: parenchymal gliogenesis (Boda and Buffo, 2010; Trotter et al., 2010), parenchymal neurogenesis (Bonfanti and Peretto, 2011) and periventricular neurogenesis (Migaud et al., 2010); little is known about non-canonical cell genesis, which seems to lack integration within the parenchyma; (iii) progenitors in both canonical and non-canonical neurogenic sites are activated in different pathological conditions (Arvidsson et al., 2002; Luzzati et al., 2011); in spite of such activation, the response to injury is substantially non-coordinated and/or abortive, not leading to effective brain repair (Kernie and Parent, 2010; Bonfanti, 2011; Bonfanti and Peretto, 2011).

**Figure 1:**
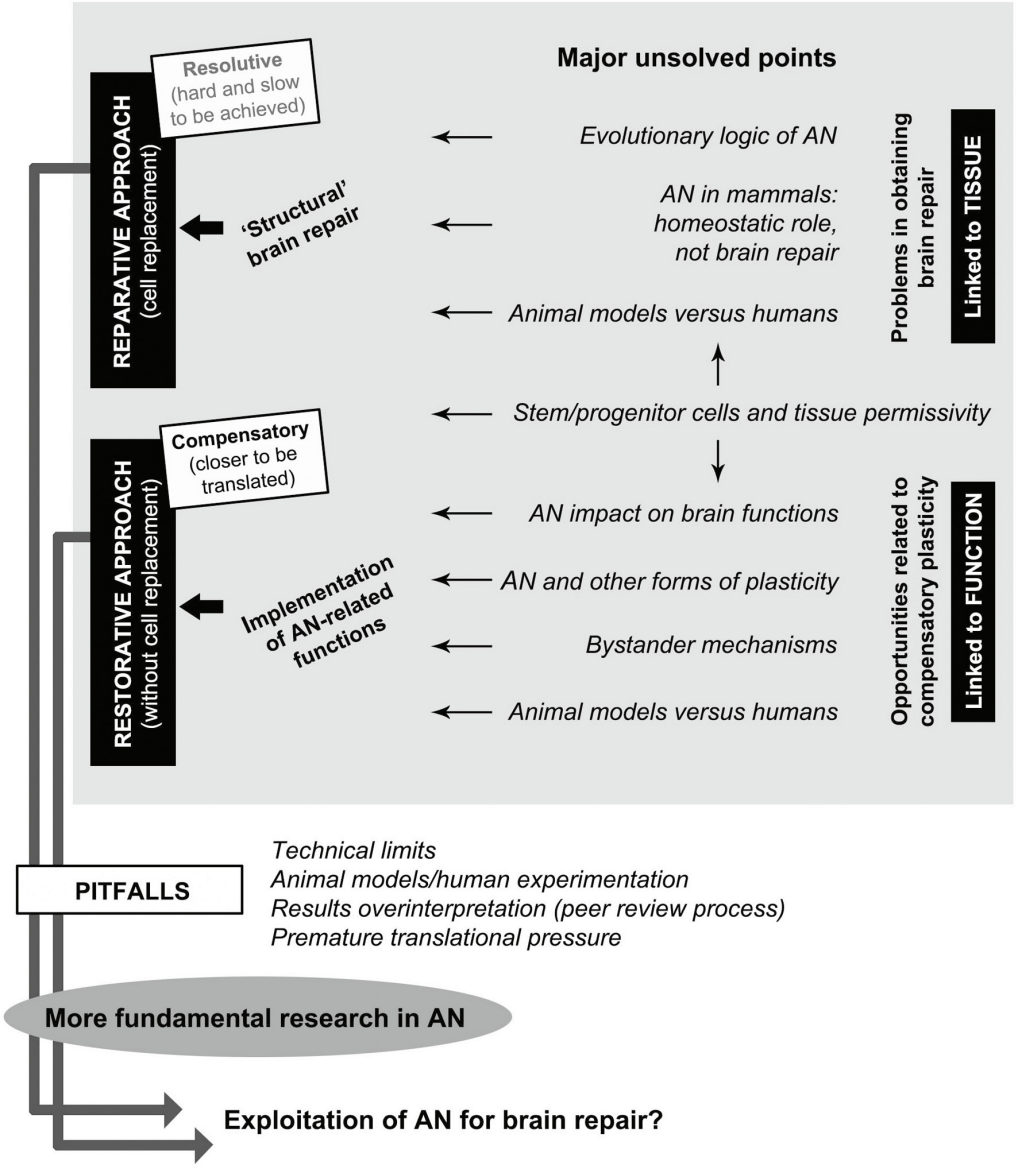
**Interplay between adult neurogenesis (AN), some of its major unsolved points, and possible perspectives for brain repair**. Gray box: among many open issues still existing in the potential role of AN in neural plasticity (gaps of knowledge), a general distinction should be made between: (i) tissue-related problems depending on evolutionary issues and hampering brain repair/regeneration (top), and (ii) function-related opportunities depending on possible homeostatic roles of AN which could be exploited/implemented for restorative approaches (bottom). Stem/progenitor cell availability and tissue permissivity (middle) are essential aspects for allowing translational perspectives to be figured out in both directions. Reparative approaches, which imply cell replacement as the ultimate goal of regenerative medicine, are not available at present. Restorative approaches include different therapeutic perspectives linked to the implementation of physiological functions of AN aimed at obtaining compensatory plasticity in the absence of cell replacement, both in the damaged and undamaged (age-related decline) brain. Successful achievement of these goals is linked to further investments in fundamental research by overcoming of current pitfalls in the AN field.

Behind these blocks of acquired knowledge large amounts of unknown facts and concepts are still hidden. First of all, AN remarkable plasticity has introduced a new kind of complexity: that of dynamic, developmental-like processes related to neuronal addition occurring within a substantially static tissue. Moreover, in mammals, the CNS is structurally, functionally, and evolutionarily refractory to repair, healing, and regeneration. These facts make it extremely challenging to exploit AN as a therapeutic tool, also because the variables involved are dependent among each other and linked by different hierarchies (Figure 1). Here, we will analyze the key points still remaining open in the AN field, considering them as potential hurdles to a full understanding of the biological process itself, and, in turn, to its possible exploitation for brain repair.

## An physiological function(s) vs. brain repair: evolutionary aspects

Unlike most vertebrates, in adult mammals spontaneous neurogenesis is primarily linked to homeostatic/physiological roles and hardly directed to repair (Bonfanti, 2011). This view is supported by many studies which addressed the issue of reactive (lesion-induced) neurogenesis, indicating “activation” of neural progenitors which substantially do not provide cell replacement, most of the newly born/mobilized cells being fated to die (Kernie and Parent, 2010; Luzzati et al., 2011).

The fact that many non-mammalian vertebrates can perform brain repair/regeneration (Endo et al., 2007; Grandel and Brand, 2013) underlines the involvement of evolutionary aspects at the developmental, anatomical, stem cell types/availability and tissue-specific environment levels (Bonfanti, 2011). The occurrence of AN in the CNS of all vertebrates suggests the naive (and wrong) view of a direct relationship between neurogenic activity and regenerative capability (Ferretti, 2011): AN is not sufficient for regeneration to occur, and other aspects should be considered. Beside lower intrinsic regenerative properties (and lower availability of stem/progenitor cells), the mammalian CNS is characterized by more detrimental tissue reactions, in fact hampering regeneration. Immune cell activation leading to inflammation is an early response after injury that is common to most animal groups. Yet, whereas in many non-mammalian vertebrates initial acute inflammation stimulates regeneration without subsequent detrimental tissue responses, in mammals neuroinflammation leads to the formation of the glial scar with consequent impairment of regeneration (Mescher and Neff, 2006; Sofroniew, 2009; Kyritsis et al., 2014). In other words, stem/progenitor cell availability alone cannot grant regenerative capacity if glial cell activation and inflammatory reactions also occur. A theory explains the failure in mammalian brain repair as a result of evolutionary constraints in which the injured CNS would not favor a strategy of regeneration, but rather one of minimizing further damage (Weil et al., 2008). Hence, important gaps of knowledge still exist, both in mammals and other vertebrates, concerning homeostastic/metabolic functions and tissue reaction aspects linked to AN, and the role of the immune system, which still remain largely unexplored (Schwartz et al., 2013). All these variables are involved in determining the differences between neurogenic and non-neurogenic tissue local environments, and, in turn, their permissivity to reparative processes.

## Progenitor cells, tissue environment, and an outcome(s)

Although cell proliferation exists throughout the intact CNS and is enhanced by several physiological/pathological conditions, it does not produce substantial neurogenic outcomes in the parenchyma out of the canonical sites (olfactory bulb, dentate gyrus). The main aspects that seem essential in granting CNS neurogenic/reparative capacity are: occurrence of specialized progenitor types and tissue permissivity. The SVZ and SGZ neurogenic niches harbor stem cells that appear very specified in their commitment (Obernier et al., 2014) and thus hard to divert toward other fates. As to parenchymal neural progenitors in non-canonical sites it is not yet clear what is their origin, nature, fate, and function(s). Yet, these cells do represent promising substrates for future research for several reasons (abundance, widespread distribution, region-specific differentiative commitment). Wherever stem/progenitor cells are located, both in canonical and non-canonical sites, unraveling the mechanisms underlying their quiescence or activation is also essential for their possible manipulation (Basak et al., 2012). Nevertheless, the functional availability of proper stem/progenitor cells is not sufficient to grant AN and repair in the absence of a receptive tissue environment: olfactory bulb and hippocampus circuits are permissive to neuronal integration, whereas the mature parenchyma allows less or no integration (Bonfanti and Peretto, 2011).

Taken together, these facts add hurdles to the ultimate goal of making mammalian AN processes useful for cell replacement. In spite of a large amount of data concerning the regulation of canonical AN (in terms of modulation; see Kempermann, 2011), very little is known about the cellular/molecular factors which allow the interaction between progenitors and the mature CNS tissue (permissivity) both in neurogenic and non-neurogenic sites. Such tissue permissivity is strictly linked to intrinsic features (adhesion molecules, extracellular matrix, availability of growth factors, etc.) which are maintained and/or delayed from development, thus allowing the AN process to persist during adulthood. In this context, a few studies have thoroughly investigated the steps that drive and regulate the shift between embryonic and AN.

## Animal models vs. humans

Most knowledge on AN has been gathered on laboratory rodents, what is a clear limit for translational approaches. Indeed, neurogenic processes differ quite among mammals as to their location, rate, niche organization, and the postnatal temporal windows in which they occur (Bonfanti and Peretto, 2011). For instance, while cell migration from SVZ to olfactory bulb persists throughout life in mice, it is exhausted very early in human infants (Sanai et al., 2011). In the human hippocampus, measures of ^14^C concentration in genomic DNA show a substantially constant rate of AN through ages, in contrast with an evident decrease in young rodents (Spalding et al., 2013). Also parenchymal neurogenesis remarkably varies among mammals, showing species-specific regional localizations (Luzzati et al., 2006; Ponti et al., 2008). Other inter-mammalian differences concern specific functions related to the ecological needs and behavioral activity of the animals (Barker et al., 2011).

Besides AN heterogeneity, mammals also differ in their brain anatomy and physiology (Carlson, 2012), and this can affect the impact AN might exert on the whole brain function (see below). Also the average lifespan varies among mammals, thus implying that differences in postnatal development of CNS areas, brain maturation, puberty, make it difficult to compare AN in different species (Lindsey and Tropepe, 2006; Kuhn and Blomgren, 2011). Hence, restraining AN research to laboratory rodents may introduce several bias in the search for translational outcomes. If comparative/evolutionary studies through phylogeny are essential to unravel the common logic of AN, the study of differences among mammals are also important for correctly interpreting/modeling the possible contribution of AN to homeostasis and brain repair in humans.

## To which extent an impacts the brain function?

Addition of newborn neurons in the olfactory bulb and hippocampus optimizes neurological functions/behaviors such as social interaction/reproduction, memory, learning, and pattern separation (Sahay et al., 2011; Feierstein, 2012). These two brain regions are essential for behavioral outputs critical for survival of the individuals and species (Mucignat-Caretta et al., 2012; Snyder and Cameron, 2012). Accordingly, AN is assumed as a mechanism which promotes life-long adaptability of individuals to environmental complexity and novelty (Freund et al., 2013). Regulation of AN is achieved through integration of multiple external and internal stimuli, which implies activity of multiple brain regions/circuits and complex feedback loops (Kempermann, 2011). Thus, though restricted to the olfactory bulb and hippocampus, AN potentially impacts diverse brain functions (Snyder and Cameron, 2012; Lepousez et al., 2013), which might explain that in mammals it occurs only in two regions. Although little is known on this hypothesis, since the anatomical, functional, molecular bases underlying the above mentioned interactions are far from being clarified, the possible impact of AN on other brain functions/circuits can have important translational implications (Leuner and Gould, 2010; Kheirbek et al., 2012; Snyder and Cameron, 2012; Quadrato et al., 2014). Several data are already available on the link between hippocampus, pattern separation/overgeneralization of sensory stimuli and anxiety disorders (Leuner and Gould, 2010; Kheirbek et al., 2012). The recent finding that human hippocampal AN appears substantially maintained during adulthood (Spalding et al., 2013) adds new interest to this issue, also in the perspective of implementing cognitive functions during aging (Bordey, 2014). Yet, proper translational outcomes imply definitive clarification of the real rate/impact of AN in humans during postnatal development and adulthood, in physiological, and pathological condition.

Finally, if AN does extensively affect the brain function(s), it should be emphasized that it is only one among other forms of CNS plasticity and that very little is known about the mechanisms which underlie their mutual relationships.

## An and other forms of plasticity and/or repair strategies

The CNS of mammals, in spite of having lost most of its regenerative/repair capacity with respect to other phyla, is endowed with different forms of structural plasticity involving pre-existing cellular elements (Bonfanti and Nacher, 2012). Among them, the most known and widespread is the experience-dependent synaptic plasticity that can occur in response to environmental enrichment and after a lesion in the form of compensatory events, i.e., synaptic formation/elimination and axonal sprouting/pruning (Brown et al., 2009; Chen and Nedivi, 2010; Fu and Zuo, 2011). Further levels of structural plasticity are found in a population of “immature,” non-newly generated neurons of the cerebral cortex (Gomez-Climent et al., 2008). These cells, in spite of their differentiated neuronal morphology (Luzzati et al., 2009), express immature neuronal markers and show very few synapses on their membrane, thus not being integrated in the adult cortical circuits, like “stand by” elements (Bonfanti and Nacher, 2012). All these forms of structural plasticity could be useful in rehabilitation approaches that mostly exploit compensatory plasticity of undamaged, preexisting structures (Dobkin, 2004). If and how all these forms of plasticity are integrated with AN is a fully open question, also taking into account that mammalian AN itself consists of heterogeneous processes involving the canonical neurogenic niches and progenitors located throughout the CNS tissue (non-canonical cell genesis; Boda and Buffo, 2010; Bonfanti and Peretto, 2011).

A better knowledge of the mutual relationships existing within the vast landscape of neural plasticity is fundamental to correctly figure out restorative therapeutic approaches in neurology (Figure 1). In recent years, several studies have started to unravel new modes of communication between stem/progenitor cells (endogenous or transplanted) and resident cells of the CNS, also involving a cross-talk with the immune system. This communication is at the basis of the so called “bystander effects,” namely a series of paracrine mechanisms which can exert beneficial effects even in the absence of cell replacement (Martino et al., 2011). An hypothesis supported by several works is that transplanted stem/progenitor cells can exert a bystander immune modulation by modifying the inhospitable microenvironment at the injury site through the release of soluble molecules such as chemokines and cytokines (Pluchino and Cossetti, 2013). More recently, it has been proposed that the same effects might be also exerted by cell mobilization/activation of endogenous stem/progenitor cells toward adjacent injured sites (Kokaia et al., 2012). In perspective, these studies have the added value of considering neural plasticity, AN, and brain repair in the context of a cross-talk between the CNS and the immune system, the latter being far more important than previously thought. Hence, the study of cell-cell interaction/paracrine communication does represent a fully open, promising field of research, aimed at developing “restorative” rather than “cell replacement” strategies.

## Some pitfalls in an field: interpretation of results and peer review process

Since the beginning, following the emphasis of a new form of CNS plasticity, the reparative potential of AN has been overestimated by the scientific community, at least under its possible regenerative outcome. This fact is reflected by statements contained in many papers dealing with both spontaneous and lesion-induced AN in which the results obtained are more or less directly linked with potential therapeutic outcomes, in the absence of direct evidence for such a link. These statements, although originally intended as “possibilities” by the authors, are frequently amplified by the media, thus generating unjustified hopes in patients affected by neurological diseases (this aspect is analyzed in Cattaneo and Bonfanti, 2014). The source of this problem is well addressed by Kerner (2006): “Many individual research reports, while suggesting exciting new innovations that may lie ahead in the future, have little or no immediate application in public health and/or clinical practice. Thus, it may be difficult for the practice community to distinguish the signal about what is currently important to practice from the noise of what may or may not become important in the future.”

In the neurological context, restorative approaches in the absence of cell replacement, including modulation of physiological/paracrine functions (Martino et al., 2011; Bordey, 2014; Quadrato et al., 2014) should always be kept clearly distinct from the true reparative/regenerative processes involving cell replacement. In the history of AN scientific publications, from the initial “naïve” belief that AN could easily represent the biological substrate for cell replacement in the CNS, to a more recent overestimation of the bystander effect therapeutic potential, it appears that too many unjustified claims actually bypass the filter of peer review. We feel that this habit is not rewarding for the public representation of science and even not for the future of AN field.

## Future perspectives: an and fundamental research

It appears evident that having introduced excessive and premature translational issues toward brain repair did not solve the problem of neurological diseases. Moreover, a simplistic view of AN as a ready-made therapeutic tool could even be counterproductive, since it might put down the interest in AN studies. In spite of some oversights along the route, the increasing knowledge gathered during the last 20 years is enormous, considering the changes that AN research has produced in our vision of brain plasticity. Non-invasive technologies are essential to study AN in humans (e.g., Spalding et al., 2013 for ^14^C detection), although further technical advancements are needed (e.g., cell imaging enhanced resolution; Sierra et al., 2011). Yet, because the AN field has become widely diversified both in its goals and lines of research (Figure 1), obtaining of future breakthroughs will require a multidisciplinary integration of high specific expertise in order to gain a whole transversal view of the variables involved (for the several meanings of translation/implementation concepts see Kerner, 2006). The current gaps of knowledge should be filled with new basic research prior to put them into a translational view. We can be confident that applied (beneficial and profitable) products of fundamental research will be eventually achieved in the future, although not visible/predictable at the time of the experimental phases (Press, 2013). Confidence in the eventual usefulness of basic research should be sufficient to bring back AN in the normal context of science.

### Conflict of interest statement

The authors declare that the research was conducted in the absence of any commercial or financial relationships that could be construed as a potential conflict of interest.
